# A Tetrad Catastrophe: Paraneoplastic Syndrome With Abducens Palsy, Intracranial Hypertension, and Optic Neuropathy in Primary Lung Cancer

**DOI:** 10.7759/cureus.67335

**Published:** 2024-08-20

**Authors:** Venushia Chandran, Nurul Ain Masnon, Rona A Nasaruddin, Jemaima Che Hamzah, Adzleen Mohmood, Andik Fadilah Abdul Aziz

**Affiliations:** 1 Ophthalmology, Faculty of Medicine, National University of Malaysia, Kuala Lumpur, MYS; 2 Ophthalmology, Hospital Kuala Lumpur, Ministry of Health Malaysia, Kuala Lumpur, MYS; 3 Ophthalmology, Kuala Lumpur Hospital, Kuala Lumpur, MYS; 4 Ophthalmology, Hospital Sultan Abdul Aziz Shah, University of Putra Malaysia, Kuala Lumpur, MYS; 5 Nuclear Medicine, National Cancer Institute, Putrajaya, MYS

**Keywords:** lung cancer, abducens palsy, optic neuropathy, intracranial hypertension, paraneoplastic syndrome

## Abstract

We report a unique case of paraneoplastic syndrome (PS) associated with primary lung cancer. A 57-year-old woman experienced headaches and bilateral visual loss one month after the onset of isolated right abducens palsy. Examination revealed bilateral poor visual acuity (VA), papilledema, and persistent right abducens palsy. Neuroimaging was normal. Lumbar puncture revealed high cerebrospinal fluid (CSF) opening pressure and protein levels. She was started on acetazolamide and pulse methylprednisolone followed by oral corticosteroids. Her abducens nerve palsy resolved, but her VA deteriorated. Anti-Hu and anti-CV2 were positive. A positron emission tomography (PET) scan revealed primary lung cancer, and she died six months after her initial presentation. This case demonstrated that PS poses a diagnostic challenge and may be associated with poor prognosis.

## Introduction

Paraneoplastic syndromes (PS) are a group of rare, cancer-associated immune-mediated disorders that occur in 7%-15% of cancer patients [1]. Clinically, PS usually arises at sites distant from the primary tumor and its metastases. PS occurrence may precede the discovery of the primary tumor or develop after a cancer diagnosis [2]. Although it can occur with any cancer, PS is most commonly associated with small-cell lung cancer, breast cancer, gynecological tumors, and hematological malignancies [3]. We describe a rare case of paraneoplastic neurological syndrome (PNS) with abducens nerve palsy, intracranial hypertension, and optic neuropathy as the presenting symptoms of lung carcinoma.

## Case presentation

A 57-year-old woman with underlying hypertension and diabetes mellitus presented with a two-week history of gradual impairment of bilateral vision associated with headache, nausea, and tinnitus. There was no fever, limb weakness, seizures, cough, hemoptysis, shortness of breath, altered bowel habits, loss of appetite, or loss of weight. She was a non-smoker and denied any family history of malignancy. She had presented with isolated right abducens nerve palsy one month prior. She was treated for ischemic right abducens nerve palsy after contrast-enhanced CT (CECT) of the brain, and inflammatory markers were unremarkable.

**Figure 1:**
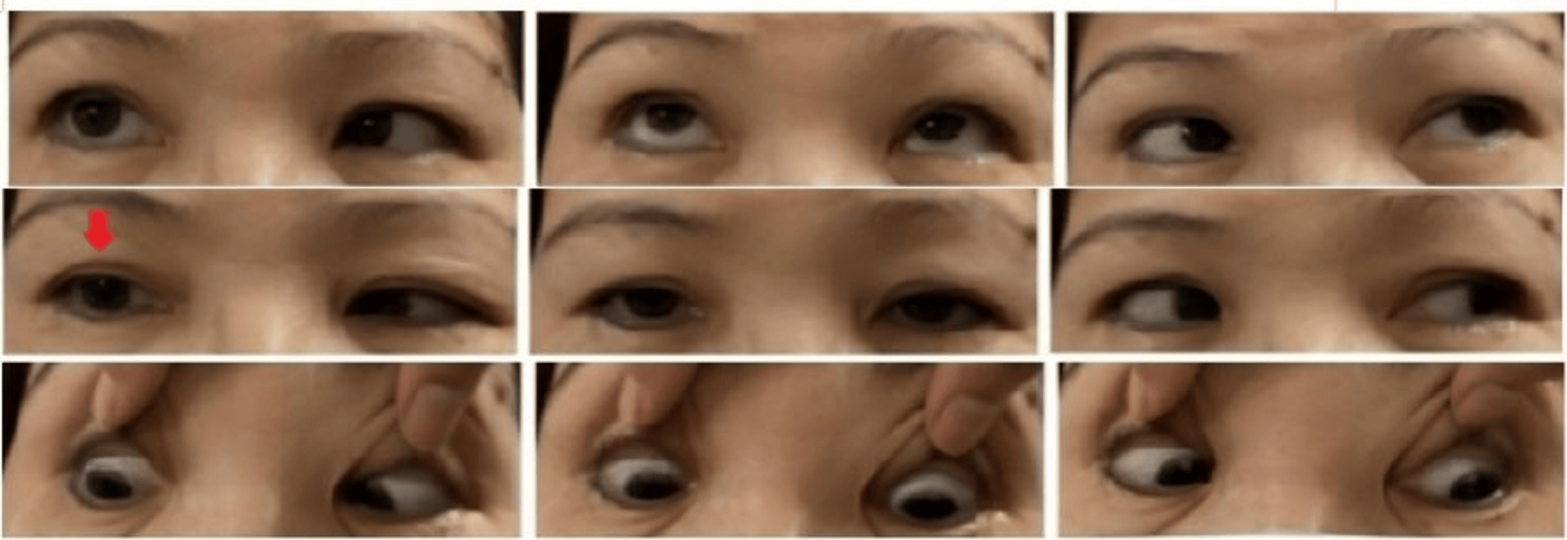
A nine-gaze photo of ocular motility showing right abducens palsy as demonstrated by limitation in abduction (red arrow)

On examination, her blood pressure was 130/85 mmHg, and her random blood glucose level was 9.5 mmol/L. Visual acuity (VA) in her right eye was 1/60 and 6/120 in her left eye. Extraocular muscle examination showed limitation in right eye abduction, demonstrating right abducens nerve palsy (Figure 1).

**Figure 2:**
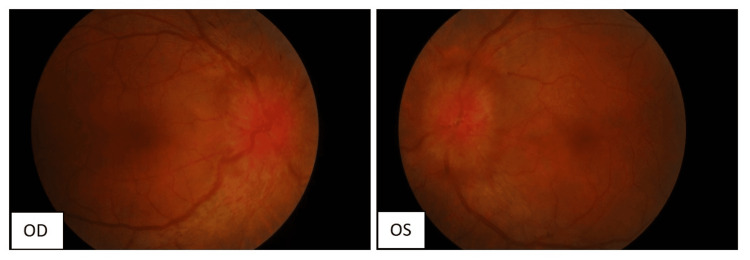
Fundus photo showing bilateral eye severe optic disk swelling with Frisen grade 5 papilledema OD: Oculus dexter (right eye); OS: Oculus sinister (left eye).

Anterior segment examination was unremarkable; however, fundoscopy revealed severe bilateral optic disk swelling (Figure 2). Other neurological and systemic examinations were normal.

Repeated CECT brain, CT venogram, MRI brain, and orbit were unremarkable. Lumbar puncture revealed a high cerebrospinal fluid (CSF) opening pressure (46 cm H_2_O), high CSF protein (812 mg/dL), and positive oligoclonal band. Otherwise, the CSF was negative for viruses, bacteria, fungi, and atypical cells. Inflammatory markers were within normal ranges. Anti-aquaporin-4 and anti-myelin-oligodendrocyte glycoprotein antibody tests were negative. Carcinoembryonic antigen (CEA) level (287 ug/L) was significantly elevated, while other tumor markers were unremarkable. Chest X-ray, abdominal ultrasound, and endoscopy of the upper and lower intestinal tract appeared unremarkable.

**Figure 3:**
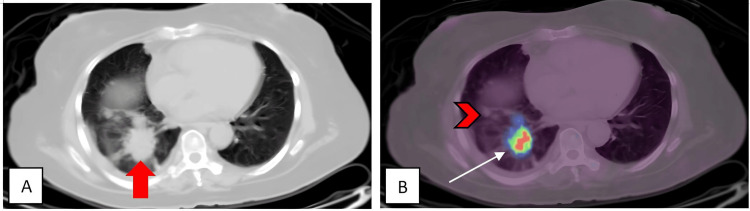
PET scan revealed a primary lesion in the lung (red arrow). (A) Axial view. (B) The white arrow shows an FDG-avid spiculated nodule at the posteromedial segment of the right lower lobe. The red arrowhead shows a few smaller satellite nodules that are non-FDG-avid. PET: Positron emission tomography; FDG: Fluorodeoxyglucose.

**Figure 4:**
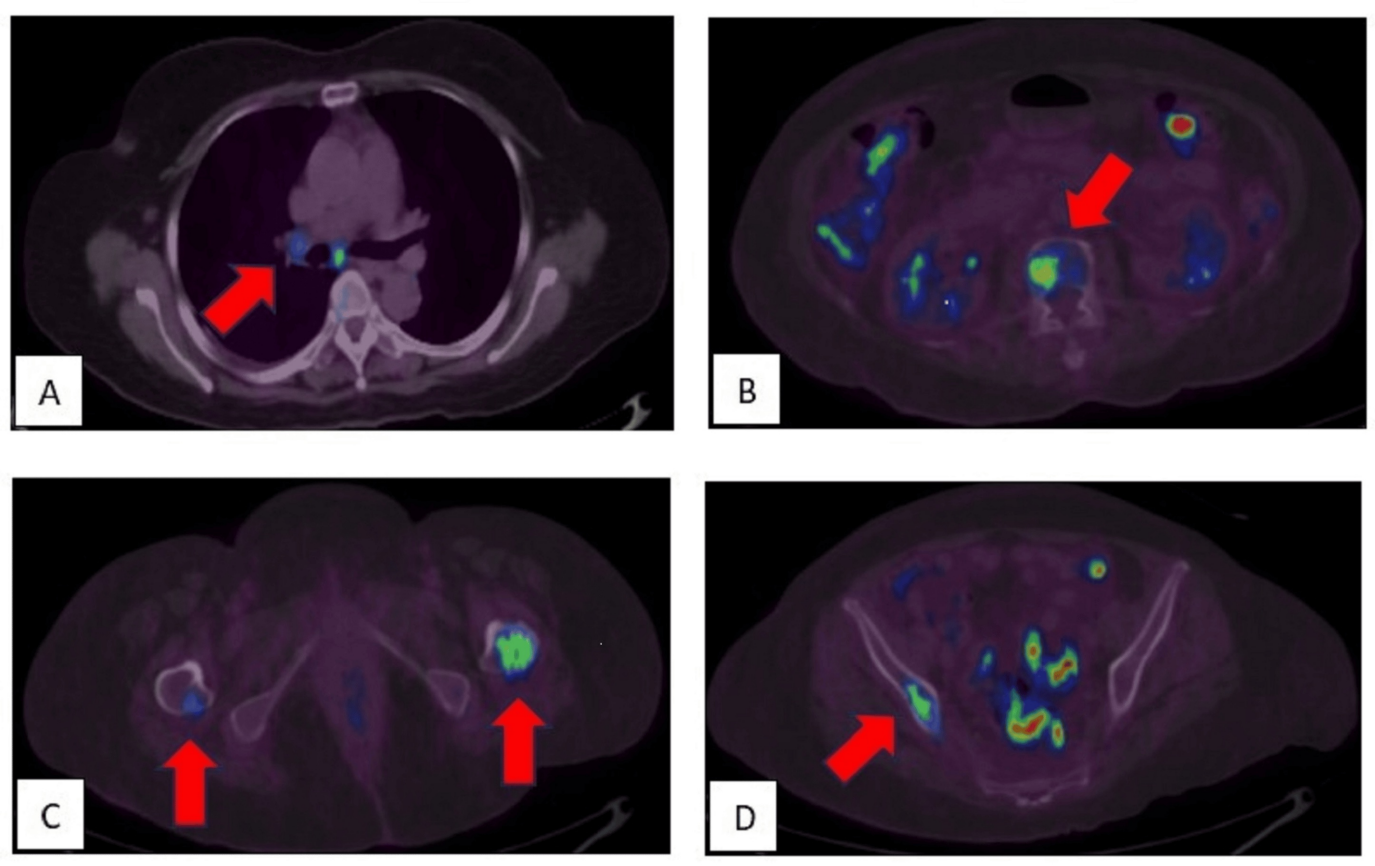
PET scan showing (A) several FDG-avid mediastinal nodes indicating regional involvement. Multiple foci of FDG-avid lesions in the bones indicate various sites of bone metastasis at (B) L2 vertebral body, (C) trochanteric regions of proximal femori, and (D) right ilium. FDG elsewhere is physiological due to bowel uptake. PET: Positron emission tomography; FDG: Fluorodeoxyglucose.

Our patient was initially treated for bilateral optic neuritis with intracranial hypertension of undetermined origin. She received oral acetazolamide 250 mg twice daily and intravenous methylprednisolone 1 gram per day for three days, followed by a course of oral prednisolone. Her headache and abducens nerve palsy improved, but her VA worsened to no light perception bilaterally. Paraneoplastic serology revealed positive results for anti-Hu and anti-CV2, and a subsequent PET scan showed primary lung malignancy (Figure 3) with regional node involvement and various bone metastasis sites (Figure 4). She refused a CT-guided biopsy and further treatment. She died six months after her initial presentation due to disease progression.

## Discussion

PS is a unique condition that may affect diverse organ systems to produce dermatological, rheumatological, endocrine, hematological, or neurological syndromes. PNS is relatively rare, affecting only 0.01% of patients with cancer, with an incidence of 0.6 per 100,000 person-years. Approximately 60%-80% of PNS cases are detected before cancer is diagnosed. Overall, it is estimated that 0.5%-1% of all patients with cancer have clinically disabling PNS [1,3,4].

The development of neurological symptoms in PNS has been linked to the aberrant production of onconeuronal antibodies by the primary tumor cells. These antibodies attack antigens expressed on neuroglial tissue throughout the central and peripheral nervous system due to cross-reaction with the specific host neuronal tissue sites, leading to pathological processes including loss of neuronal plasticity and apoptotic neuronal damage [5-7].

Our patient initially presented with isolated right abducens nerve palsy, followed by subacute bilateral visual loss and symptoms of elevated intracranial pressure. Various PNS manifestations have been reported, including opsoclonus, myoclonus, encephalomyelitis, limbic encephalitis, cerebellar ataxia, and sensory neuropathy, but these were not present in our patient [1]. The occurrence of intracranial hypertension as part of PNS is rare and has been associated with non-small-cell lung carcinoma, gastric signet ring cell carcinoma, duodenal cancer, and acute promyelocytic leukemia [7]. Research has shown that intracranial hypertension symptoms and ocular motility deficits may improve, but paraneoplastic optic neuropathy may lead to severe visual loss, as observed in our patient.

She had normal neuroimaging that did not correlate to the extent of her presentation, which was consistent with other PS cases and warranted further investigation. High CSF opening pressure with evidence of high protein and an oligoclonal band in the CSF indicate an ongoing inflammatory process, and CEA was noted to be significantly elevated in our patient. Although CEA is commonly an important marker for colorectal cancer, it can also arise in other cancers, including lung cancer [8]. Anti-Hu and anti-CV2 are PNS markers that are most commonly associated with small-cell lung carcinoma. These were noted to be positive, and the PET scan finally revealed the presence of primary lung cancer with multiple sites of bone metastasis. PET scans are superior to conventional imaging for the detection of primary and metastatic lesions because PET uses fluorodeoxyglucose, a radioactive isotope that is taken up by actively growing and metabolically demanding cancerous cells, which may reveal the occult primary malignancy site and metastases [9,10].

Treatment of the underlying malignancy if detected at an early stage may offer a better outcome in PNS. Long-term immunosuppression, plasmapheresis, and intravenous immunoglobulin have also been reported as potential treatments for PNS, but results are variable, and PNS may lead to permanent neurological disability [9]. Our patient died six months after her initial ophthalmic presentation due to the rapid progression of her underlying malignancy with poor survivals being documented in more advanced lung cancers.

## Conclusions

Patients who present with atypical optic neuropathy with other neurological involvement and normal neuroimaging should raise a high index of suspicion of PS disorder after ruling out autoimmune and infective causes. Thorough investigation including tumor markers, paraneoplastic markers and radioimaging will help in detecting the underlying cause and aid in detecting malignancies early. Late detection may be fatal as in the case of of our patient who has had bone metastasis.
